# Kaempferol enhances intestinal repair and inhibits the hyperproliferation of aging intestinal stem cells in *Drosophila*

**DOI:** 10.3389/fcell.2024.1491740

**Published:** 2024-10-10

**Authors:** Liusha Zhao, Ting Luo, Hong Zhang, Xinxin Fan, Qiaoqiao Zhang, Haiyang Chen

**Affiliations:** ^1^ Center of Gerontology and Geriatrics and Laboratory of Stem Cell and Anti-Aging Research, National Clinical Research Center for Geriatrics and State Key Laboratory of Respiratory Health and Multimorbidity, West China Hospital, Sichuan University, Chengdu, Sichuan, China; ^2^ Department of Gastroenterology and Hepatology and Laboratory of Inflammatory Bowel, West China Hospital, Sichuan University, Chengdu, Sichuan, China; ^3^ Department of Gastroenterology, Affiliated Hospital of North Sichuan Medical College, Nanchong, Sichuan, China; ^4^ Frontiers Science Center for Disease-Related Molecular Network, State Key Laboratory of Respiratory Health and Multimorbidity and National Clinical Research Center for Geriatrics, West China Hospital, Sichuan University, Chengdu, Sichuan, China

**Keywords:** Kaempferol, intestinal stem cell, ROS, *Drosophila*, aging

## Abstract

**Introduction:**

Intestinal stem cells (ISCs) are crucial for tissue repair and homeostasis because of their ability to self-renew and differentiate. However, their functionality declines significantly with age, resulting in reduced tissue regeneration and a higher risk of age-related diseases. Addressing this decline in ISC performance during aging presents a substantial challenge. The specific impact of nutrients or dietary elements on ISC adaptive resizing is urgent to explore.

**Methods:**

*Drosophila* ISCs are an ideal model for studying development and aging because of their genetic richness, ease of manipulation, and similarity to mammalian tissues. As the primary mitotically active cells in the *Drosophila* gut, ISCs are flexible in response to dietary and stress signals. Manipulating signaling pathways or dietary restrictions has shown promise in regulating ISC functions and extending lifespan in flies, these approaches face broader applications for aging research.

**Results:**

Kaempferol is well-regarded for its antioxidant, anti-inflammatory, and potential anticancer effects. However, its impacts on ISCs and the associated mechanisms remain inadequately understood. Our findings indicate that Kaempferol accelerates gut recovery after damage and improves the organism’s stress tolerance. Moreover, Kaempferol suppresses the hyperproliferation of aging ISCs in *Drosophila*. Further investigation revealed that the regulatory effects of Kaempferol on ISCs are mediated through the reduction of endoplasmic reticulum (ER) stress in aging flies and the modulation of excessive reactive oxygen species (ROS) levels via ER-stress pathways. Furthermore, Kaempferol exerts regulatory effects on the insulin signaling pathway, thereby contributing to the attenuation of ISC senescence.

**Discussion:**

This study reveals that Kaempferol promotes intestinal homeostasis and longevity in aging flies by targeting ER stress and insulin signaling pathways, though the exact molecular mechanisms require further exploration. Future research will aim to dissect the downstream signaling events involved in these pathways to better understand how Kaempferol exerts its protective effects at the molecular level.

## Introduction

Intestinal stem cells (ISCs) play a critical role in gut repair, aging support, and the maintenance of homeostasis, owing to their proliferative capacity and self-renewal abilities (de Morree and Rando, 2023; Ermolaeva et al., 2018). However, their functionality declines significantly with age, resulting in reduced tissue regeneration and a higher risk of age-related diseases (Brunet et al., 2023; Ermolaeva et al., 2018; Lopez-Otin et al., 2023). Addressing this decline in ISC performance during aging presents a substantial challenge. *Drosophila* ISCs are an ideal model for studying development and aging because of their genetic richness, ease of manipulation, and similarity to mammalian tissues (Jasper, 2020). As the primary mitotically active cells in the *Drosophila* gut, ISCs show considerable flexibility in response to dietary and stress signals. They can divide symmetrically to produce more ISCs or asymmetrically to generate progenitor cells, such as EnteroBlasts (EBs), EnteroEndocrine cells (EEs), or large polyploid EnteroCytes (ECs) (Guo and Ohlstein, 2015). Although manipulating signaling pathways or dietary restrictions has shown promise in regulating ISC functions and extending lifespan in animal models, these approaches face practical limitations for broader application. Additionally, the specific impact of nutrients or dietary elements on ISC adaptive resizing is still not well understood (Du et al., 2020; Fan et al., 2023; Fang et al., 2019; Kim et al., 2022; Qin et al., 2024; Yan et al., 2022).

Kaempferol, a naturally occurring flavonoid prevalent in fruits and vegetables, emerges as a strong candidate for dietary interventions (Gidaro et al., 2016). Its widespread dietary availability, minimal toxicity, cost-efficiency, and ease of daily incorporation make it well-suited for extensive use (Gidaro et al., 2016; Yang et al., 2023). Numerous studies have demonstrated kaempferol’s wide-ranging pharmacological benefits, including anticancer (Lee and Kim, 2016; Luo et al., 2012; Luo et al., 2011; Shrestha et al., 2021), anti-inflammatory (Wang et al., 2020), anti-obesity (Han et al., 2021), antiviral (Bangar et al., 2023; Gao et al., 2023), antioxidant (Guo et al., 2015; Wang et al., 2018), immune-modulatory (Bangar et al., 2023), and neuroprotective effects (Dong et al., 2023; Holland et al., 2020; Yuan et al., 2021). However, the specific effects of kaempferol on ISC aging and injury, along with its underlying mechanisms, remain inadequately explored. During the processes of aging and injury, reactive oxygen species (ROS) and endoplasmic reticulum (ER) stress can adversely affect ISC function and adaptability (Du et al., 2021; Yan et al., 2022). Given kaempferol’s capacity to neutralize ROS, reduce inflammation, and provide anticancer benefits (Guo et al., 2015; Han et al., 2021; Lee and Kim, 2016; Luo et al., 2012; Luo et al., 2011; Wang et al., 2018; Wang et al., 2020). We aim to investigate its potential to slow ISC aging and enhance damage recovery in *Drosophila*.

In this research, we demonstrate that Kaempferol serves as a potent natural compound by effectively curbing ISC hyperproliferation during aging. Notably, Kaempferol extends the lifespan of aged *Drosophila* by regulating gut function and sustaining homeostasis. Mechanistically, it mitigates ISC hyperproliferation by reducing ER stress through the upregulation of UPRER-related genes. Additionally, Kaempferol controls the excessive accumulation of ROS via ER-stress signaling and concurrently suppresses the insulin signaling pathway, contributing to delayed aging. Overall, we have identified Kaempferol as a novel natural phenolic compound that slows ISC aging and offers protective effects against damage.

## Results

### Kaempferol inhibits the hyperproliferation of ISCs in aging *Drosophila*

Recent research has identified certain molecules with anti-aging properties in *Drosophila*, which have also shown similar benefits in mammals (Du et al., 2020; Fang et al., 2019; Wu et al., 2022). Building on these insights, we aimed to identify compounds in vegetables and fruits that could potentially extend lifespan. Several vegetable and fruit extracts have been reported to have such effects on *Drosophila*. Among these, Kaempferol, a flavonoid-rich in vegetables and fruits, drew our attention due to its well-documented health benefits and disease-preventing capabilities (An and Kim, 2015; Bangar et al., 2023; Dong et al., 2023; Gao et al., 2023; Gidaro et al., 2016; Guo et al., 2015; Han et al., 2021; Holland et al., 2020; Lee and Kim, 2016; Luo et al., 2012; Luo et al., 2011; Shrestha et al., 2021). To evaluate Kaempferol’s potential to inhibit ISC aging, we employed the *esg*-GFP/CyO reporter line, where green fluorescent protein (GFP) is regulated by the escargot (*esg*) gene (Korzelius et al., 2014), to assess its impact on ISC proliferation.

**FIGURE 1:**
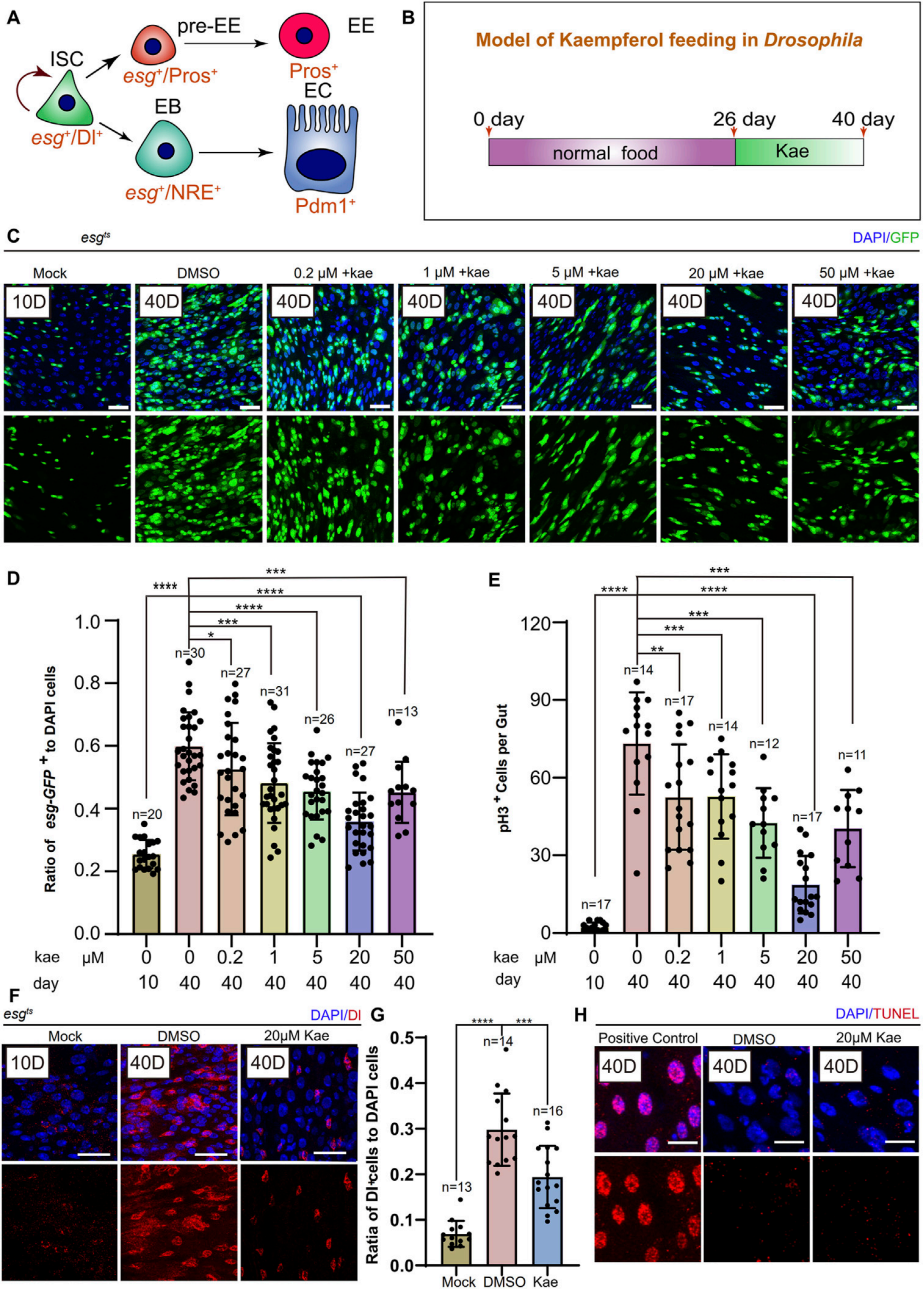
Kaempferol Modulates the hyperproliferation of ISCs in a concentration-dependent manner during aging. **(A)** ISC division and differentiation model: ISCs (marked as Dl^+^ and esg^+^) undergo symmetric division for self-renewal and asymmetric division to produce enteroendocrine progenitor cells pre-EEs (marked as esg^+^ and Pros^+^) or EBs (marked as esg^+^ and NRE^+^). Pre-EEs differentiate into EEs (marked as Pros^+^), while EBs develop into ECs (marked as Pdm1^+^). **(B)** Model of Kaempferol feeding in aging *Drosophila*: Mock refers to flies hatched for 10 days and fed a standard diet. Kae indicates flies given Kaempferol. **(C)** Model of Kaempferol feeding in aging *Drosophila*: “Mock” denotes flies hatched for 10 days and fed a standard diet. “Kae” refers to flies treated with Kaempferol. **(D)** The proportion of esg-GFP^+^ cells to DAPI-stained cells per region of interest (ROI) in *Drosophila* midguts treated with varying concentrations of Kaempferol (0.2, 1, 5, 20, and 50 µM) compared to untreated controls. **(E)** The number of pH3^+^ cells per gut in 40-day-old *Drosophila* midguts, treated with varying concentrations of Kaempferol (0.2, 1, 5, 20, and 50 µM) or without treatment. **(F)** Representative images of immunofluorescence staining showing ISCs in *Drosophila* midguts, treated and untreated with 20 µM Kaempferol. Nuclei stained with DAPI (blue) Dl (red) marked ISCs. The top images represent the merged images and the bottom images represent ISCs. **(G)** The proportion of Dl^+^ cells to DAPI^+^ cells per ROI without or with 20 µM Kaempferol treatment. **(H)** Representative immunofluorescence images of ISCs in *Drosophila* midguts, with or without 20 µM Kaempferol treatment. Nuclei are stained with DAPI (blue), and ISCs are labeled with Dl (red). The top images show merged images; the bottom images highlight ISCs. Scale bars denote 25 μm **(C, F, H)**. Error bars show SD. Statistical significance was assessed with Student’s t-tests: *p < 0.05, **p < 0.01, ***p < 0.001; ns indicates *p* > 0.05.

Previous research has indicated that in young *Drosophila*, ISCs either self-renew or differentiate into ECs and EEs (Guo and Ohlstein, 2015) (Figure 1A). With aging, These cells undergo aberrant proliferation and differentiation, leading to the excessive proliferation of ISCs and progenitor cells (Jasper, 2020). To examine this, we first fed *Drosophila* normal food for 26 days, then supplemented their diet with different concentrations of Kaempferol (0.2, 1.5, 20, 50 μM) for 14 days (Figures 1B, C). We assessed the number of ISCs and progenitor cells marked by *esg*-GFP^+^, pH3^+^ (a proliferation marker), and Dl^+^ (an ISC marker). Flies treated with 20 μM Kaempferol had fewer *esg*-GFP^+^, pH3^+^ (Figures 1C–E), and Dl^+^ cells (Figures 1F, G) compared to those fed diets added DMSO. At 50 μM, Kaempferol’s ability to reduce age-related ISC hyperproliferation diminished, suggesting potential side effects at higher concentrations (Figures 1C–E). TUNEL staining confirmed that the reduced ISC proliferation was not due to apoptosis of *esg*-GFP^+^ cells (Figure 1H). We also examined the impact of Kaempferol on ECs (marked with pros^+^) and EEs (marked with NRE^+^), which showed similar effects in aged flies (Supplementary Figures S1A–D). Overall, our data indicate that Kaempferol exerts a potent inhibitory effect on ISC hyperproliferation and attenuates gut hyperplasia in aged *Drosophila*.

### Kaempferol promotes the repair of the intestine under injury conditions and improves stress tolerance in *Drosophila*

**FIGURE 2:**
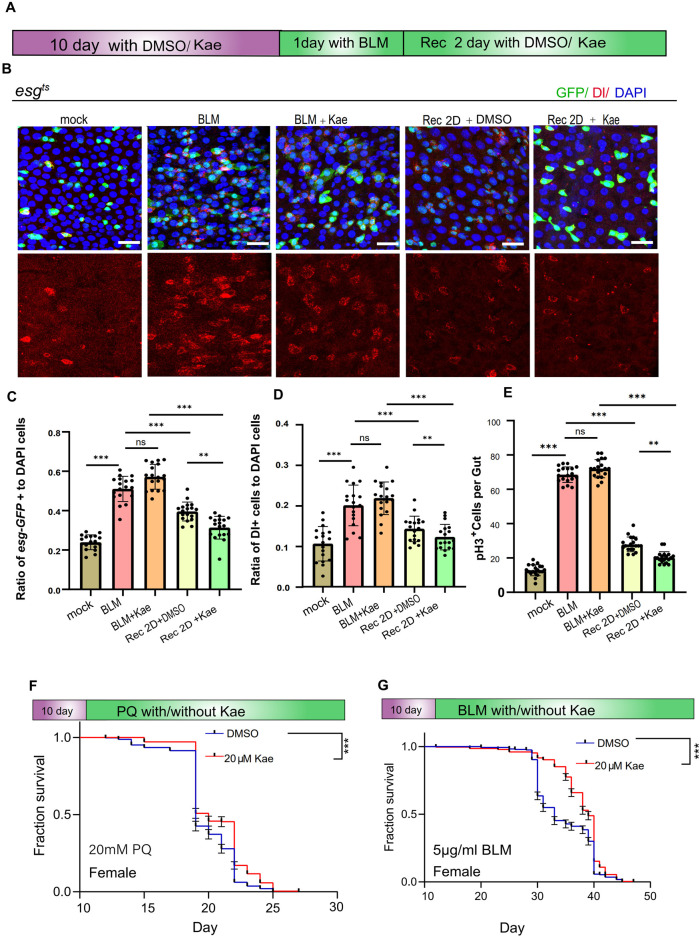
Kaempferol promotes injury repair in the intestinal and enhances healthspan under injury conditions. **(A)** Model of Kaempferol feeding in *Drosophila* during BLM treatment. **(B)** Immunofluorescence images of *Drosophila* midguts fed with 25 μg/mL BLM, with or without the addition of 20 µM Kaempferol. Nuclei are stained with DAPI (blue), GFP highlights ISCs and progenitor cells (green), and Dl identifies ISCs (red). The top images show merged images, the middle images highlight ISCs and progenitor cells, and the bottom images focus on ISCs. **(C)** The proportion of esg-GFP^+^ cells to DAPI^+^ cells per ROI in midguts treated with BLM, with or without Kaempferol supplementation. **(D)** The proportion of DI^+^ cells to DAPI^+^ cells per ROI in midguts treated with BLM, with or without Kaempferol supplementation. **(E)** The number of pH3^+^ cells per fly gut, with or without 20 µM Kaempferol, under PQ/BLM treatment conditions. **(F, G)** Survival rates of female wild-type flies treated with DMSO (blue curve) or Kaempferol (red curve) under 20 mM PQ **(F)** or 5 μg/mL BLM **(G)** conditions. Data from three independent experiments are shown.

Previous studies have indicated that *Drosophila* midguts undergo continuous turnover and can regenerate after tissue damage (Lucchetta and Ohlstein, 2012). Chemical damage, such as from bleomycin (BLM), triggers ISC proliferation. Given Kaempferol’s positive effects on *Drosophila* gut health (Amcheslavsky et al., 2009), we examined whether it could enhance the gut’s resistance to injury. After a 1-day BLM treatment, the flies were placed on normal food for a 2-day recovery (Figure 2A). Surprisingly, 2 days post-injury, *Drosophila* treated with Kaempferol showed a more significant reduction in ISC numbers (marked by *esg* and Dl) and pH3^+^ cells compared to controls, indicating that Kaempferol supports intestinal repair and limits ISC hyperproliferation under stress (Figures 2B–E). Additionally, Paraquat (PQ), known to induce oxidative stress by increasing ROS production, is commonly used to cause intestinal damage in *Drosophila* (Szafrańska et al., 2016). Kaempferol notably extended the lifespan of both female and male *Drosophila* exposed to PQ/BLM treatment (Figures 2F, G; Supplementary Figures S2A, B). In conclusion, these findings imply that Kaempferol safeguards ISCs from damage while improving *Drosophila*’s resilience to stress.

### Kaempferol prevents intestinal dysfunction and extends lifespan in aging *Drosophila*

**FIGURE 3:**
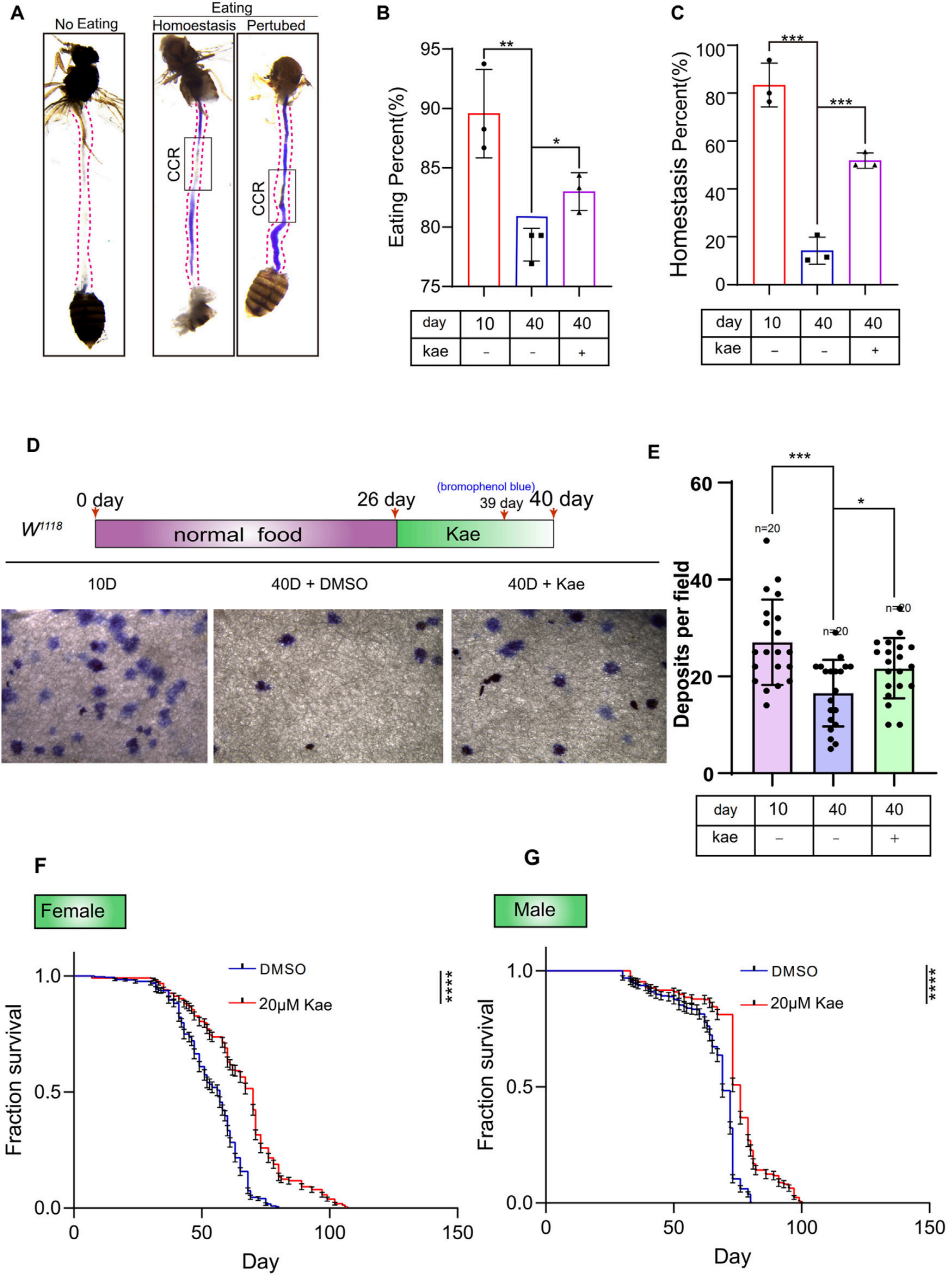
Kaempferol maintains gut homeostasis and extends the lifespan of *Drosophila*. **(A)** Images of intestinal acid-base regulation and non-feeding intestines. The black box highlights the CCR. “Non-eating” indicates wild-type flies that did not consume food, with the CCR shown in white. “Eating” refers to flies consuming bromophenol blue. “Homeostasis” shows the CCR in yellow, while “Perturbed” depicts it in blue. **(B)** The percentage of eating intestines in this experiment **(A)**. Data from three independent experiments are shown. **(C)** The percentage of homeostasis intestines in this experiment **(A)**. Data from three independent experiments are shown. **(D)** Representative images of waste deposits in flies treated with or without Kaempferol. Three separate experiments were conducted. **(E)** Deposits per field in experiment **(D)**. Results are obtained from three independent experiment data. **(F, G)** Survival rates of female and male wild-type flies with DMSO (blue line) or Kaempferol (red line) treatment starting at 10 days old. Results are based on three independent trials.

Ensuring the intestinal barrier remains intact is essential for upholding epithelial balance, shielding against pathogens, and supporting immune tolerance to beneficial bacteria (Gervais and Bardin, 2017; Naszai et al., 2015). The decline in ISC functionality and genetic integrity is a key factor in the deterioration of tissue function with aging (Biteau et al., 2008; Gervais and Bardin, 2017; Naszai et al., 2015). Since Kaempferol has shown promise in preventing age-related and injury-induced ISC decline, we further examined its potential to protect intestinal functions in *Drosophila* during aging. To assess this, we analyzed the copper cell region (CCR) condition in both young and old *Drosophila*. The CCR’s function decreases with age, disrupting intestinal acid-base balance (Li et al., 2016). In the *Drosophila* midgut, the CCR secretes acid, which is detected using bromophenol blue, a pH indicator. When intestinal homeostasis deteriorates and acid secretion decreases, the CCR remains blue. In contrast, if acid secretion improves and homeostasis is restored, the CCR turns yellow. Our results indicate that Kaempferol supplementation effectively maintains food intake and intestinal acid-base balance in aged *Drosophila* (Figures 3A–C). Additionally, Kaempferol improves intestinal function and enhances excretion in these flies (Figures 3D, E). Considering previous research and our findings that highlight the importance of intestinal regeneration for lifespan extension (Biteau et al., 2010), we also investigated Kaempferol’s impact on lifespan. Our data show that Kaempferol feeding extends lifespan in both male and female *Drosophila* (Figures 3F, G). In summary, Kaempferol supplementation counteracts the decline in intestinal function associated with aging and enhances the lifespan of *Drosophila*.

### Kaempferol inhibits the hyperproliferation of ISCs through ER-stress response

**FIGURE 4:**
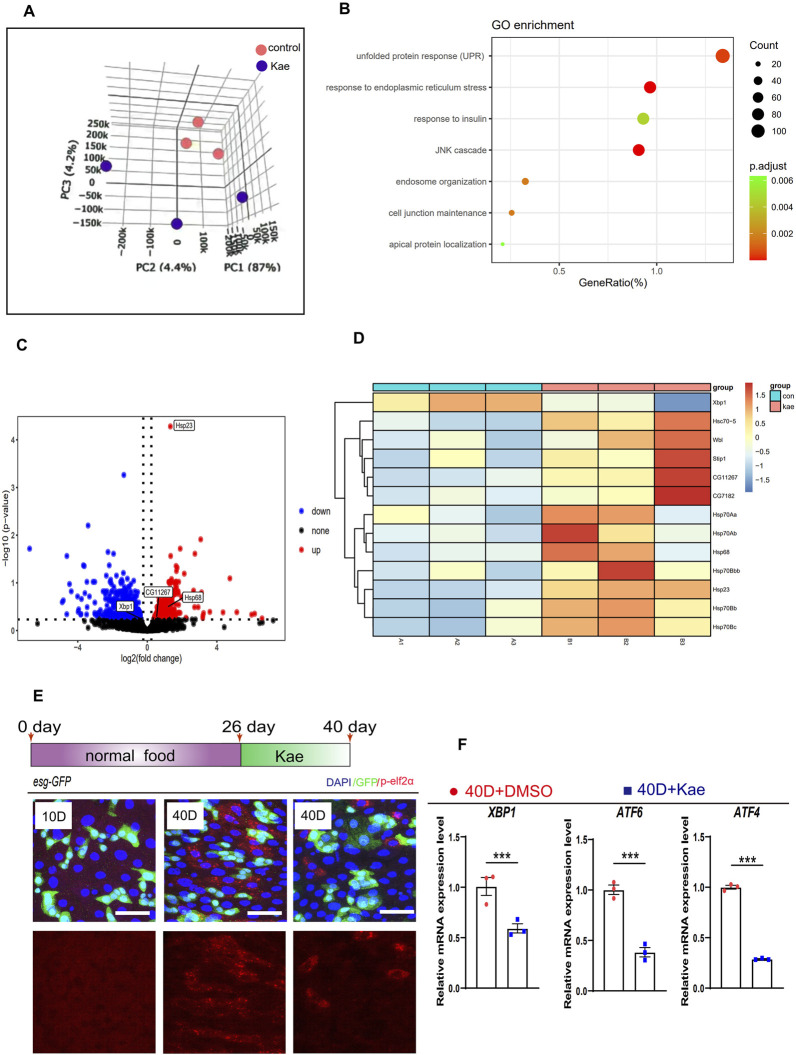
Kaempferol inhibits ISC hyperproliferation through the ER-stress signaling. **(A)** PCA of midguts from 40-day-old wild-type flies: one cohort was treated with 20 µM Kaempferol, and the other with DMSO only. **(B)** GO pathway enrichment analysis was performed on upregulated and downregulated genes in a pair-wise comparison between 40-day-old *Drosophila* midguts treated with or without Kaempferol. **(C)** The volcano plot displays genes that are differentially expressed in 40-day-old *Drosophila* midguts with Kaempferol treatment compared to those without. Red dots highlight genes that are remarkably upregulated, blue dots mark genes that are remarkably downregulated, and gray dots indicate genes with no significant change. **(D)** The differential gene expression of UPRER in midguts of flies with Kaempferol feeding compared to the control group is shown in the heatmap. **(E)** Representative immunofluorescence images of p-eIF2α staining in midguts with or without Kaempferol treatment. The top images show merged images, while the bottom images focus on p-eIF2α signals. Three independent experiments were conducted. **(F)** RT-qPCR was used to measure the mRNA levels of ER-stress-related genes in the midguts of 40-day-old wild-type *Drosophila*, comparing those with and without Kaempferol treatment (administered starting at 26 days). Three independent experiments were conducted.

The proliferation of ISCs is tightly controlled by gene expression (Guo and Ohlstein, 2015). To understand how Kaempferol influences ISC proliferation, we conducted the RNA-seq on midguts from flies treated with Kaempferol compared to controls. Principal component analysis (PCA) highlighted significant differences between the two groups (Figure 4A). Gene Ontology (GO) enrichment analysis indicated a dramatic increase in genes associated with the unfolded protein response (UPR) in flies treated with Kaempferol (Figure 4B). The UPRER refers to a subset of UPR genes activated by ER stress, which occurs when the ER becomes overwhelmed with misfolded proteins (Rath et al., 2012). To suppress ER stress, the UPR triggers its target genes to alleviate the accumulation of unfolded proteins by enhancing the production of several stress-responsive chaperones (Du et al., 2021; Hetz, 2012). GO enrichment analysis revealed significant differences in UPRER expression between Kaempferol-treated and control flies. We analyzed URPER-related genes and created a volcano plot to highlight the significance and fold changes of differentially expressed genes. The results showed that UPRER genes, such as *Hsp23* and *HSP68*, were upregulated in the midguts of 40-day-old wild-type flies treated with Kaempferol compared to controls (Figure 4C). Heatmap analysis further identified UPR target genes (*Hsp70Bbb*, *Hsp70Ab*, *HSC70-5*, *Hsp70Bb*, *Hsp68*, *Hsp70Bc*, *Hsp23*, and *Hsp70Aa*) with markedly higher expression levels in the aged midguts of *Drosophila* treated with Kaempferol compared to untreated flies (Figure 4D). The UPRER is known to be crucial in regulating ISC hyperproliferation associated with aging (Du et al., 2021). Drawing on prior research and our current data, we propose that Kaempferol addresses aging-related gut hyperplasia through pathways involving UPRER genes. Moreover, we analyzed the expression levels of p-eIF2α, an essential transcription factor in the ATF4 ER stress pathway (Mao et al., 2019). Phosphorylated eIF2α (p-eIF2α) serves as a reliable biomarker for evaluating ER stress levels in cells (Rath et al., 2012), In *Drosophila* ISCs, p-eIF2α was significantly reduced (Figure 4E). This finding suggests that Kaempferol likely mitigates ISC aging by alleviating ER stress, as evidenced by the increased expression of UPRER target genes in aged flies treated with Kaempferol. Immunostaining further demonstrated that Kaempferol effectively lowered elevated p-eIF2α levels in these cells (Figure 4E). To validate Kaempferol’s effect on ER-stress signaling, we conducted RT-qPCR analyses, which revealed decreased levels of ER-stress-related genes (Ko and Brandizzi, 2024; Kumar and Maity, 2021). ER-stress-related genes (*ATF6*, *ATF4*, and *XBP1*) were reduced in the guts of aged *Drosophila* treated with Kaempferol (Figure 4F). These results suggest that Kaempferol may affect ISC proliferation by modulating ER-stress signaling.

### Kaempferol suppresses the hyperproliferation of ISCs partly through its antioxidant function in aged *Drosophila*

**FIGURE 5:**
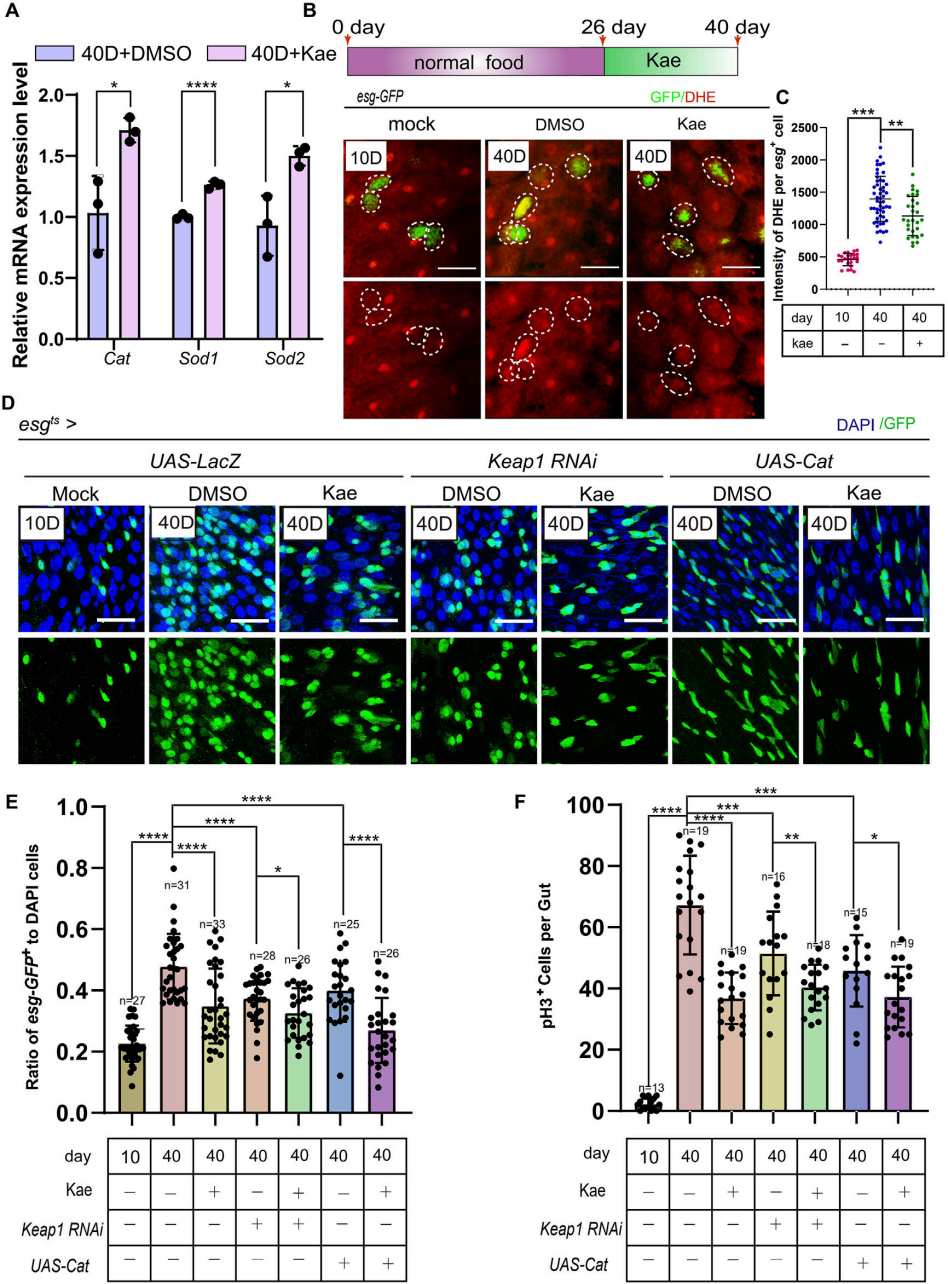
Kaempferol prevents ISC hyperproliferation through its antioxidative functions during aging. **(A)** RT-qPCR utilized to measure the mRNA levels of ROS-related genes (sod1, sod2 and Cat) in the midguts of 40-day-old wild-type *Drosophila*, comparing those with and without Kaempferol treatment (administered starting at 26 days). Three independent experiments were completed. **(B)** Representative images of DHE staining are provided. esg-GFP^+^ cells (green) and DHE staining (red) are highlighted by white dashed lines. The top images show merged images, while the bottom images focus on DHE staining. DHE fluorescence intensity was measured in esg-GFP^+^ cells, with each dot representing a single cell. **(C)** Quantitation of DHE fluorescence intensity in esg-GFP^+^ cells from this experiment. **(D)** Kaempferol further inhibited ISC proliferation in Uas-LacZ, Cat-overexpressed, and Keap1-depleted aged ISCs (starting from day 26). The top images show merged images, and the bottom images highlight esg^+^ cells, labeled with GFP. **(E)** The proportion of esg-GFP^+^ cells to DAPI^+^ cells per ROI in experiment **(C)**. **(F)** The number of pH3^+^ cells per gut in experiment **(C)**.

To further understand Kaempferol’s impact on aging ISCs, we reviewed existing studies, revealing that Kaempferol can inhibit antioxidant activity (Wang et al., 2018; Wang et al., 2020). It also impacts the expression of genes involved in ER-stress signaling (Figures 4B–D, F). ER stress is closely linked to ROS accumulation (Uddin et al., 2021). ER stress can lead to ROS production through mitochondrial DNA damage. Connecting ROS to mitochondrial stress as a consequence of ER signaling (Salminen and Kaarniranta, 2010). To investigate whether Kaempferol’s antioxidant effects are preserved in *Drosophila*, we measured the expression of antioxidant-related genes via RT-qPCR. Results showed increased levels of *Catalase* (*Cat*), *Sod1*, and *Sod2* in aged *esg* ^+^ cells treated with Kaempferol (Figure 5A). Additionally, Using the fluorescent probe DHE, we evaluated ROS levels in *esg* ^+^ cells. The results indicated a dramatic decrease in ROS in aged flies that received Kaempferol (Figures 5B, C). To examine Kaempferol’s role in ISC homeostasis, we overexpressed *Cat* and used *Keap1* RNAi in *esg* ^+^ cells with a temperature-sensitive system (*esg* ^*ts*^), as *Cat* and *Keap1* are key regulators of intracellular redox balance (Hochmuth et al., 2011). This approach revealed that *Cat* overexpression and *Keap1* RNAi reduced ROS levels and ISC hyperproliferation, as shown by fewer *esg* ^+^ and pH3^+^ cells in 40-day-old *Drosophila* (Figures 5D–F). Importantly, Kaempferol also decreased ISC proliferation in *Drosophila* with *Cat* overexpression and *Keap1* depletion (Figures 5D–F), suggesting its role extends beyond antioxidant activity to regulating ISC homeostasis.

### Kaempferol maintains ISC homeostasis partly by inhibiting the insulin signaling pathway

**FIGURE 6:**
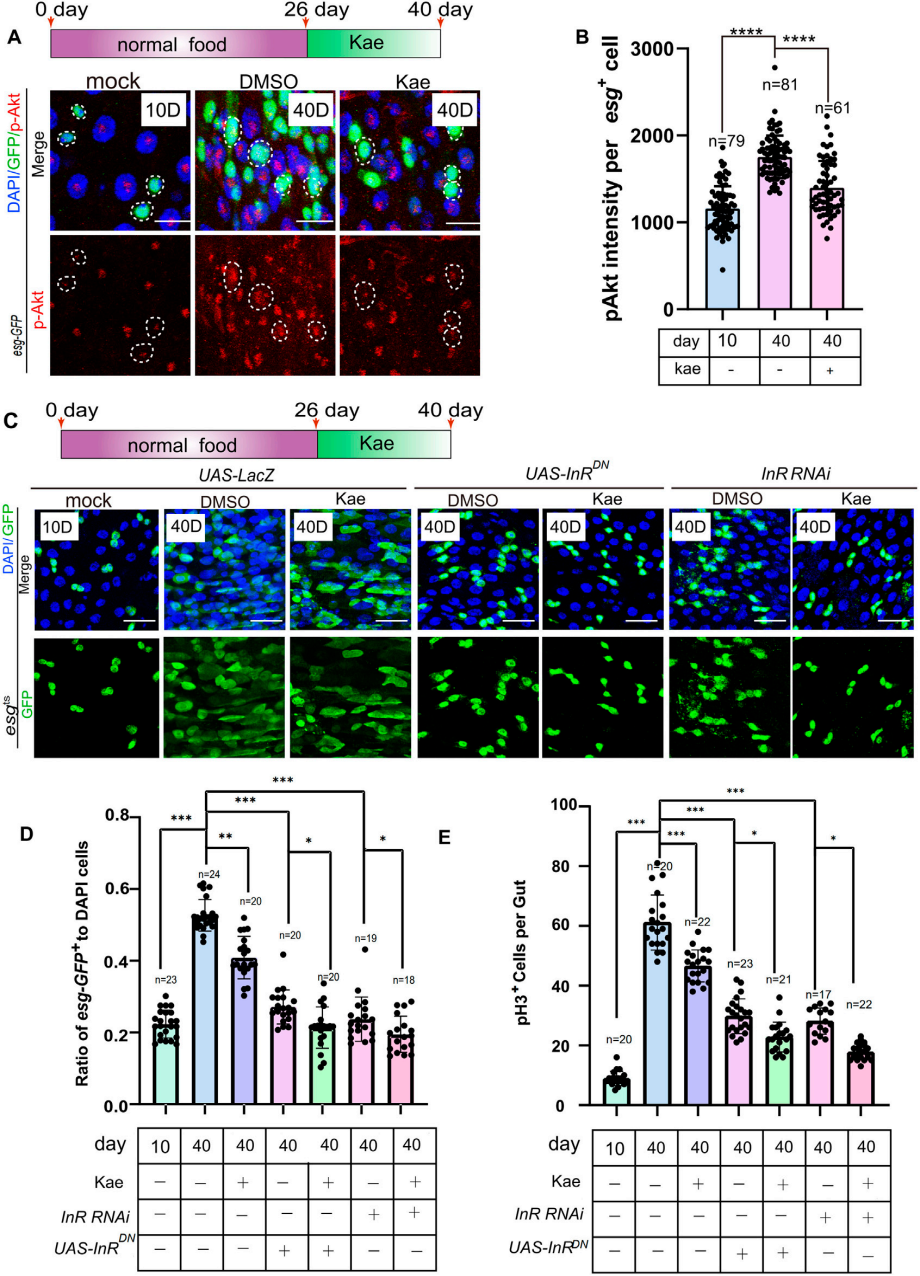
Kaempferol inhibits the hyperproliferation of ISCs in aging via the insulin signaling pathway. **(A)** p-AKT staining (red) in aged esg-GFP^+^ cells of *Drosophila* midguts, visualized through immunofluorescence imaging, comparing samples with and without Kaempferol treatment. The top images show merged images and the bottom images display p-AKT signals. **(B)** Quantitation of p-AKT fluorescence intensity in esg-GFP^+^ cells. Each dot indicates one esg-GFP^+^ cell. **(C)** Immunofluorescence images of ISCs in UAS-LacZ, UAS-InRDN, and InR RNAi *Drosophila* midguts, with or without Kaempferol treatment (starting from day 26). The top images show merged images, while the bottom images display esg^+^ cells. **(D)** The proportion of esg-GFP^+^ cells to DAPI^+^ cells per ROI in experiment **(C)**. **(E)** The number of pH3^+^ cells per fly gut.

Kaempferol’s ability to further inhibit ISC proliferation in *Drosophila* with *Keap1* RNAi and *Cat* overexpression suggests it might engage other pathways in its mechanism. Although lifespan varies greatly among species, fundamental genetic pathways that control longevity are conserved (Green et al., 2022; Guo et al., 2016; Hartl, 2016; Jasper, 2020; Melzer et al., 2020). A well-known example is the insulin/IGF-1 signaling (IIS) pathway (Giannakou and Partridge, 2007; Partridge, 2001). RNA-seq data show that Kaempferol significantly affects insulin signaling (Figure 4B). This pathway contains the insulin/IGF-1 receptor tyrosine kinase, and the serine/threonine kinase AKT (Giannakou and Partridge, 2007; Serrano et al., 2009). To investigate the impact of Kaempferol on this pathway, we measured insulin signaling activity by assessing levels of phosphorylated Akt (pAKT). Immunostaining results indicated that Kaempferol decreased pAKT levels in ISCs of aged flies (Figures 6A, B). Further validation was performed by inhibiting the insulin pathway using either *InR* RNAi (insulin-like receptor) or a dominant-negative (DN) form of *InR*, leading to a reduction in *esg*-GFP^+^ and pH3^+^ cells compared to controls (Figures 6C–E). Kaempferol supplementation also reduced these cell populations in aged flies, likely due to its antioxidant properties related to ER stress. These discoveries indicated that Kaempferol supports ISC homeostasis in aging *Drosophila* by partially inhibiting the insulin signaling pathway.

### Kaempferol suppresses ISC proliferation through the insulin and ROS signaling pathways

**FIGURE 7:**
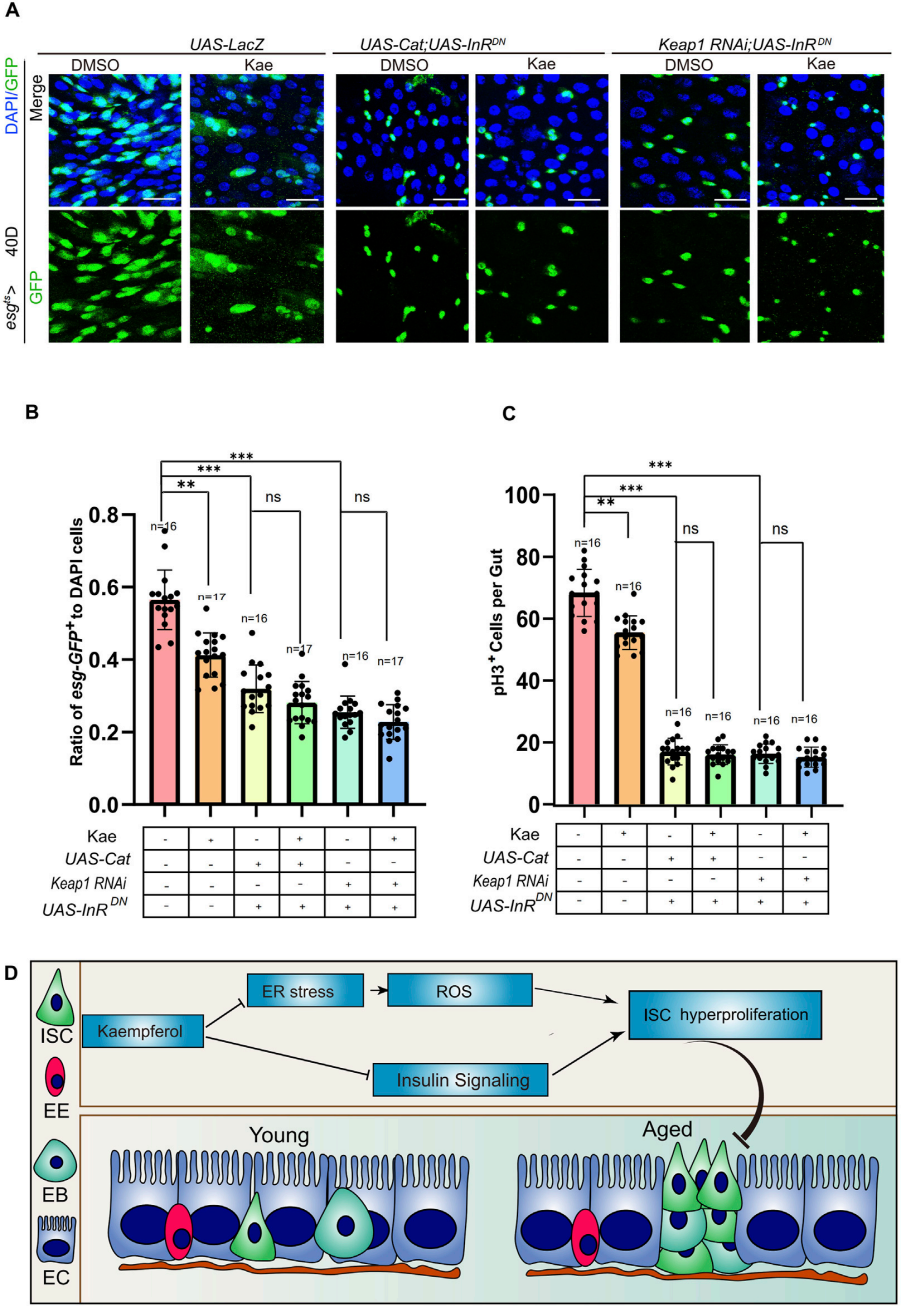
Kaempferol regulates ROS and insulin signaling pathways to inhibit the hyperproliferation of aging ISC. **(A)** Immunofluorescence images of ISCs in UAS-LacZ, UAS-Cat overexpressed, UAS-InRDN, and Keap1RNAi with UAS-InRDN *Drosophila* midguts, with or without Kaempferol treatment (administered from day 26). The top images display merged images, and the bottom images show esg^+^ cells. **(B)** The proportion of esg-GFP^+^ cells to DAPI^+^ cells per ROI in experiment **(A)**. **(C)** The number of pH3^+^ cells per fly gut **(A)**. **(D)** Model of Kaempferol suppresses ISC hyperproliferation via insulin signaling pathway conjunction with ER-stress and ROS.

As mentioned, ER stress produces ROS in mitochondria through mitochondrial DNA damage (Forgie et al., 2022). This process highlights the link between ER stress and mitochondrial stress. To explore how Kaempferol influences proliferation via ROS and insulin signaling, we conducted experiments involving *Keap1* RNAi or *Cat* overexpression, combined with the expression of a DN form of *InR* to suppress the insulin signaling pathway in 40-day-old ISCs. The findings indicated a notable reduction in both *esg*-GFP^+^ and pH3^+^ cells in these flies when compared to the control group (Figures 7A–C). Interestingly, Kaempferol did not increase these effects any further (Figures 7A–C). These results indicated that Kaempferol suppresses age-related hyperproliferation ISC by modulating both the insulin and ROS signaling pathways (Figure 7D).

## Discussion

In recent years, researchers in injury repair and aging have concentrated on understanding the underlying mechanisms and devising advanced treatments to improve tissue repair and combat age-related diseases. A key area of interest has been the effect of natural substances on tissue damage and aging. Kaempferol, a compound found in various fruits, vegetables, and dietary supplements, has garnered attention for its potential health benefits (Bangar et al., 2023). Its neuroprotective, antioxidant, anti-inflammatory, anti-cancer, and immune-modulatory properties have been documented in both *in vitro* and *in vivo* studies, showing promise against conditions like stroke, Alzheimer’s, and cancer (Dong et al., 2023; Gao et al., 2023; Gidaro et al., 2016; Guo et al., 2015; Han et al., 2021; Lee and Kim, 2016; Luo et al., 2012; Luo et al., 2011; Wang et al., 2018; Wang et al., 2020). Our research further emphasizes Kaempferol’s role in repairing ISC damage and reducing ISC hyperproliferation related to aging. We found that Kaempferol effectively mitigates ISC dysfunction, prevents gut hyperplasia, preserves intestinal PH balance, and enhances food consumption and excretion in aged *Drosophila*. Additionally, Kaempferol supplementation significantly improved ISC recovery from injury and increased survival rates of *Drosophila* under stress.

Our investigation into the mechanisms of Kaempferol revealed its ability to mitigate ER stress, regulate ROS levels in ISCs, and influence insulin signaling. The ER plays a vital role in eukaryotic cells, managing protein synthesis, folding, and transport. Disruptions to ER function can lead to an accumulation of unfolded or misfolded proteins, which causes ER stress and activates the UPR to reestablish cellular equilibrium (Bhattarai et al., 2020). Aging affects ER chaperones and folding enzymes, impairing proteostasis and causing an accumulation of misfolded proteins. Ineffective UPR activation worsens ER stress by hindering the removal of these proteins (Uddin et al., 2021). Moreover, aging is associated with increased ER stress and elevated ROS levels (Salminen and Kaarniranta, 2010). ROS, reactive molecules derived from oxygen, serve as signaling agents in normal physiological processes but can cause oxidative stress when present in excess, contributing to aging and associated diseases (Yan et al., 2022).

Kaempferol affects ISC aging by modulating the IIS pathway, which is conserved from *Drosophila* to mammals and regulates numerous physiological functions, including growth, metabolism, reproduction, stress responses, and aging (Giannakou and Partridge, 2007). The *Drosophila* genome includes eight insulin/IGF-like peptide paralogs (Post et al., 2019). Studies show that decreased IIS can extend lifespan across various species, such as worms, *Drosophila*, mice, and humans. Meanwhile, increased IIS is associated with a shorter lifespan and a higher risk of age-related diseases (Giannakou and Partridge, 2007; Post et al., 2019). Moreover, the IIS pathway is vital for stem cell homeostasis, with *Drosophila* insulin-like peptides, which act through insulin receptors, being crucial for the regulation of stem cell proliferation (Li and Geng, 2010).

Aging leads to defects in ISCs across various tissues, impairing their ability to repair damage and maintain homeostasis (Brunet et al., 2023; Ermolaeva et al., 2018). Our study reveals that Kaempferol inhibits excessive ISC proliferation by modulating ROS and insulin signaling pathways during aging. However, the specific molecular mechanisms through which Kaempferol influences these pathways in aging ISCs and tissue repair require further investigation. It is possible that Kaempferol directly interacts with certain targets to regulate these pathways. Additional research is needed to clarify Kaempferol’s precise role in ISC function and its potential effects on ISC differentiation in the context of aging.

## Conclusion

In summary, Kaempferol is a potent dietary agent with significant anti-aging effects and promotes tissue repair. It extends the lifespan and health span of *Drosophila*, offering a range of health benefits for aging individuals through several mechanisms.

## Materials and methods

### Materials availability

Any unique or stable reagents and materials discussed in this study can be synthesized using the protocols provided in the Materials and Methods section or can be requested from the lead contact.

### *Drosophila* stocks

*Drosophila* was maintained at 25°C with a 12-h light-dark cycle in controlled incubators and was provided with standard cornmeal/yeast medium. For conditional expression studies, the flies were initially kept at 18°C. To activate temperature-sensitive transgenes, flies were Switched to 29°C for the necessary period; otherwise, they were aged at 25°C. Only mated females were used for midgut studies.

### Kaempferol and bleomycin

Dissolution of Kaempferol in Dimethyl sulfoxide (DMSO) and added to the normal food medium. Female flies, collected within 3 days of eclosion, were evenly distributed into tubes containing the diet enriched with Kaempferol. The control diet was prepared with the same volume of DMSO.

A4 paper was cut into strips measuring 3 cm × 5 cm and soaked in a 25 μg/mL solution of bleomycin (Aladdin, B107423). Flies were first hungered for 2 h in empty tubes, then placed in tubes containing the bleomycin solution. After 24 h, the flies were moved to new tubes with food supplemented with 20 μmol/L Kaempferol, while DMSO was used as a control.

### *Drosophila* stocks and fly genetics

All *Drosophila* genotypes and their sources used in this study are listed in Supplementary Table S4.

### Lifespan and survival experiments

In the lifespan study, 100 mated wild-type females and 100 males of the same genetic background were separated 10 days after eclosion and distributed into five tubes, each with about 20 males and 20 females. Each tube contained food supplemented with either 20 mM PQ (Aladdin, M106760) and 5 µg BLM (Aladdin, B107423), added with 20 mM Kaempferol (MCE, HY-14590) or DSMO. Sacrificed flies were counted and recorded every 2 days, and the food in each vial was also replaced every 2 days. The experiment was conducted three times to confirm the results’ reliability and reproducibility.

For the Kaempferol lifespan study, 100 mated wild-type females and 100 males of the same genetic background were placed into five tubes, each containing around 20 males and 20 females, 3 days after eclosion. The control group was fed with food mixed with DMSO, while the experimental group received Kaempferol-mixed food. Sacrificed flies were counted and recorded every 2 days, and the food in each vial was also replaced every 2 days. The experiment was conducted three times to confirm the results’ reliability and reproducibility.

### RNA-seq

Total RNA was extracted from 30 midguts dissected from wild-type female *Drosophila* for RNA sequencing. The samples were immediately frozen in liquid nitrogen and processed by Shenzhen Chengqi Biotechnology Co., LTD (China). Sequencing was performed on the Illumina NovaSeq 6000 platform (San Diego, US) with 150 bp paired-end reads, yielding over 20 million reads per sample. Initial quality checks of the raw FASTQ files were done using FastQC (v0.11.9, http://www.bioinformatics.babraham.ac.uk/projects/fastqc/). The processed reads were aligned to the *Drosophila* reference genome (Ensembl build BDGP6, https://support.illumina.com/sequencing/sequencing_software/igenome.html), Gene symbols were annotated based on the *Drosophila* BDGP6 genome from Ensembl. Differential gene expression was assessed using DESeq2 (v1.26.0) with default parameters, focusing on genes with an absolute log2 fold change exceeding 0.5 between modified (e.g., RNAi) and control samples, and a *p*-value of less than 0.05. Pathway analysis was performed with a cluster Profiler.

### RNA purification and RT-qPCR

One hundred dissected midguts from 100 wild-type female *Drosophila* were placed in 4°C DEPC-PBS. The samples were then incubated in 1 mg/mL elastase (Sigma, cat. no. E0258) in DEPC-PBS at 25°C, with gentle mixing every 15 min. After incubation, the samples were centrifuged at 600 × *g* for 15 min at 4°C and resuspended in cold DEPC-PBS. They were then filtered through 70 µm filters and sorted using a FACS Aria II Sorter (BD Biosciences, USA). Three biological replicates were performed. For subsequent analyses, samples were lysed with a lysis buffer, and total RNA was extracted using the Cell Total RNA Isolation Kit (FOREGENE, RE-03111) following the manufacturer’s instructions. Reverse transcription was performed with oligo dT using the PrimeScript RT Reagent Kit (Vazyme, R312-01), and the first-strand cDNA was diluted tenfold with water for real-time PCR analysis (Vazyme, Q711-02). Expression levels were quantified using the 2^−ΔΔCT^ method and normalized to RpL15, with the control sample’s expression set to Primer sequences for qPCR are listed in Supplementary Table S3.

### TUNEL assay

Dissected midguts of the flies were placed in cold PBS and fixed in 4% paraformaldehyde for 30–35 min. They were then washed twice times with 0.1% PBST (PBS with Triton X-100), each wash lasting 10 min. Apoptosis was detected using the colorimetric TUNEL Apoptosis Assay Kit (Beyotime, C1098) following the instructions.

### DHE staining

The midguts of adult female *Drosophila* should be immersed in PBS after dissection and incubated with 20–30 μM DHE (MCE, HY-D0079) in the dark for 5 min. After washing the samples three times with PBS, they were immediately imaged using confocal microscopy. Signal intensities in the intestinal epithelium were analyzed with LAS-X software, and cells were identified based on fluorescence.

### Immunofluorescence microscopy for midguts

Dissected midguts of adult female *Drosophila* deposited in cold PBS. After dissection, remove the PBS and add a mixture of 4% paraformaldehyde and n-heptane (1:1) for 30 min at 25°C (room temperature). After fixation, the samples were washed twice with methanol for 5 min each. They were then subjected to three washes with PBS containing 0.1% Triton, each wash lasting 10 min. Then, overnight incubation of the midguts at 4°C was performed using primary antibodies diluted in wash buffer. The primary antibodies used in this study were listed in Supplementary Table S5.

The next day, the midguts were subjected to three washes with 0.1% PBST, each lasting 10–15 min. Subsequently, incubated with secondary antibodies and DAPI (Sigma) for 2–3 h at 25°C (Alexa 488 and Alexa 568, Invitrogen) were used at a 1:2,000 attenuation. Confocal imaging was conducted using a Leica TCS-SP8 microscope, and the results were analyzed by LAS X software.

### Fluorescence intensity statistics

Confocal microscopy was used to examine immunofluorescence images. Fluorescence intensity was measured from z-stacks using LAS X software. Images were captured with a Leica TCS-SP8 microscope and analyzed using Leica Application Suite X, Adobe Illustrator, Photoshop, and ImageJ.

### Bromophenol blue treatment

To categorize *Drosophila* midguts, 200 µL of 2% Bromophenol blue was first added to the food tubes (using a pipette tip to pierce the food, creating holes to ensure thorough absorption). After a 2-h fasting period, the flies were switched into the tubes for 24 h. The flies were then promptly dissected to capture images.

### Fly excretion measurement

200 µL of 2% Bromophenol blue was first added to the food tubes (using a pipette tip to pierce the food, creating holes to ensure thorough absorption). A4 paper strips (3 cm × 5 cm) were rolled and placed inside. Flies were hungered for 2 h, then exposed to the solution for 24 h. Afterward, the paper was imaged and analyzed for deposits.

### Software availability

R version 3.5.3 for RNA-seq analysis is available at R Project. The custom ImageJ for quantifying immunofluorescence can be accessed at ImageJ. Prism 7.0 (GraphPad), used for data analysis in this study, is available on the GraphPad website.

## Data Availability

The datasets presented in this study can be found in online repositories. The names of the repository/repositories and accession number(s) can be found in the article/Supplementary Material.

## Supplementary material

SUPPLEMENTARY TABLE S1 The top 30 elevated and decreased genes of UPR in the Kaempferol supplement compared to the control in Figure 4.

SUPPLEMENTARY TABLE S2 The genes are mainly studied UPR in Figure 4.

SUPPLEMENTARY TABLE S3 Primers used for expression analyses and the corresponding gene names.

SUPPLEMENTARY TABLE S4
*Drosophila* strains utilized in this study.

SUPPLEMENTARY TABLE S5 The primary antibodies listed in this study.
