# Supplementary figures and images for: Kaempferol enhances intestinal repair and inhibits the hyperproliferation of aging intestinal stem cells in Drosophila
Source: Front Cell Dev Biol. 2024 Oct 10;12:1491740. doi: 10.3389/fcell.2024.1491740 (PMC11499188; doi:10.3389/fcell.2024.1491740)

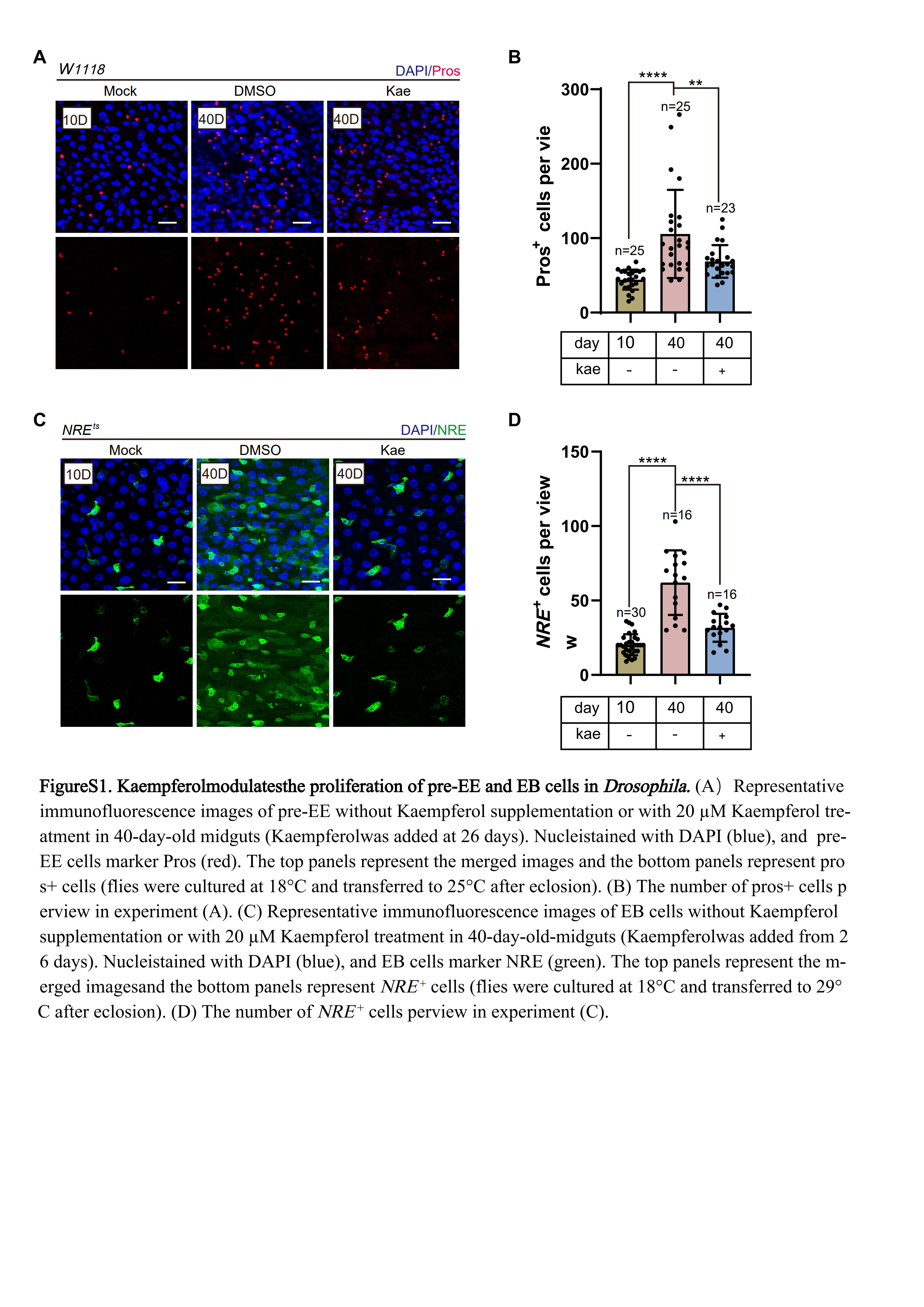

Supplement: Supplementary file 2 [file Image1.JPEG]

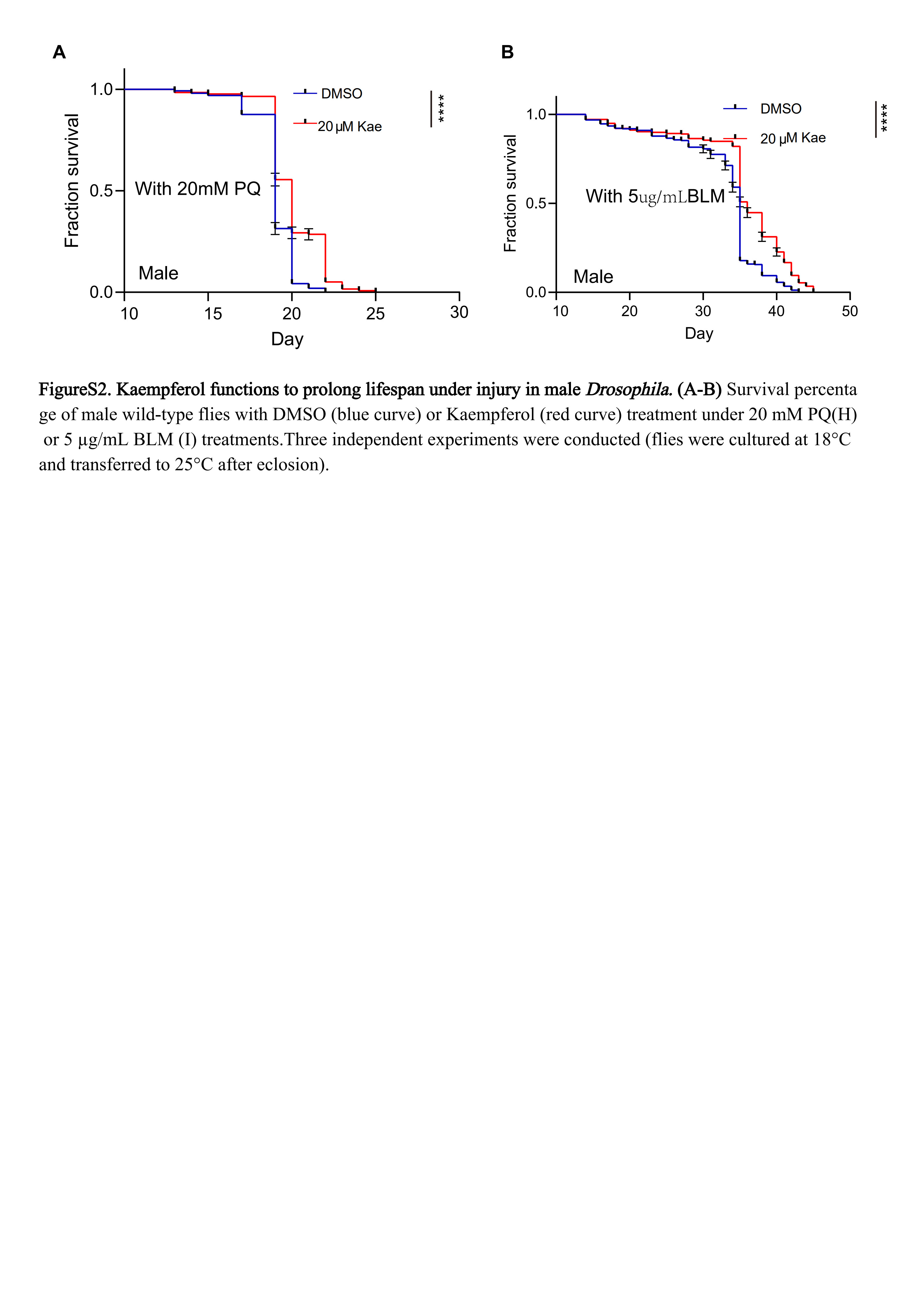

Supplement: Supplementary file 3 [file Image2.JPEG]
